# Comparative transcriptional analysis of satellite glial cell injury response

**DOI:** 10.12688/wellcomeopenres.17885.1

**Published:** 2022-05-19

**Authors:** Sara Elgaard Jager, Lone Tjener Pallesen, Lin Lin, Francesca Izzi, Alana Miranda Pinheiro, Sara Villa-Hernandez, Paolo Cesare, Christian Bjerggaard Vaegter, Franziska Denk

**Affiliations:** 1 Wolfson Centre for Age-Related Diseases, Institute of Psychiatry, Psychology and Neuroscience, King's College London, Guy's Campus, London, UK; 2 Department of Biomedicine, Danish Research Institute of Translational Neuroscience - DANDRITE, Nordic-EMBL Partnership for Molecular Medicine, Aarhus University, Aarhus C, Denmark; 3 Department of Biomedicine, Aarhus University, Denmark & Steno and Diabetes Center, Aarhus, Denmark; 4 NMI Natural and Medical Sciences Institute at the University of Tübingen, Tübingen, Germany

**Keywords:** Glia cells, satellite glial cells, single cell RNA sequencing, pain, dorsal root ganglia, nerve injury, cholesterol biosynthesis

## Abstract

**Background:** Satellite glial cells (SGCs) tightly surround and support primary sensory neurons in the peripheral nervous system and are increasingly recognized for their involvement in the development of neuropathic pain following nerve injury. SGCs are difficult to investigate due to their flattened shape and tight physical connection to neurons *in vivo* and their rapid changes in phenotype and protein expression when cultured *in vitro*. Consequently, several aspects of SGC function under normal conditions as well as after a nerve injury remain to be explored. The recent advance in single cell RNA sequencing (scRNAseq) technologies has enabled a new approach to investigate SGCs.

**Methods:** In this study we used scRNAseq to investigate SGCs from mice subjected to sciatic nerve injury. We used a meta-analysis approach to compare the injury response with that found in other published datasets.  Furthermore, we also used scRNAseq to investigate how cells from the dorsal root ganglion (DRG) change after 3 days in culture.

**Results:** From our meta-analysis of the injured conditions, we find that SGCs share a common signature of 18 regulated genes following sciatic nerve crush or sciatic nerve ligation, involving transcriptional regulation of cholesterol biosynthesis. We also observed a considerable transcriptional change when culturing SGCs, suggesting that some differentiate into a specialised *in vitro* state while others start resembling Schwann cell-like precursors.

**Conclusion: **By using integrated analyses of new and previously published scRNAseq datasets, this study provides a consensus view of which genes are most robustly changed in SGCs after injury. Our results are available via the Broad Institute Single Cell Portal, so that readers can explore and search for genes of interest.

## Introduction

Satellite glial cells (SGCs) are found in peripheral ganglia, where they tightly envelop each neuronal cell body in defined SGC-neuron units. With their flattened morphology, only ~20μm away from the neuronal soma, they are ideally located to communicate with neurons and provide a protective homeostatic microenvironment ^1– 9^.

Several studies have investigated the responsiveness of SGCs in rodent models of nerve injury, where the peripheral axonal branch is damaged through e.g. ligation, transection or crush. Despite such neuronal injury being induced at a substantial distance from SGCs in the dorsal root ganglia (DRG), it clearly has a knock-on effect on their function ^10– 13^. To date, SGC reactivity has mainly been studied with focus on changes in ATP signalling between neurons and SGCs, a decrease in K ^+^ buffering capacity, and an increase in the number of SGC-SGC gap junctions. Thus, somata of injured neurons are believed to release ATP in an action potential dependent manner, activating P2Y4, P2X7 and/or P2Y12 receptors on SGCs. This, in turn, modulates feedback signalling and, ultimately, the excitability of neurons ^9, 14, 15^. A decreased K ^+^ buffering of SGCs is thought to be driven by a reduced expression of the Kir4.1 channel ^16– 18^. This likely contributes to an increased concentration of extracellular K ^+^ within the SGC-neuron unit and thereby increases neuronal excitability ^9^. Finally, changes are observed in SGC-SGC gap junction connectivity, with a rise in the expression and functional assembly of connexin43 ^19– 24^. While such increased gap junction connectivity has been shown to be important for facilitating the spread of Ca ^2+^ waves ^24^, the functional consequences of this communication remain unclear.

Beyond injury, relatively little is known about the basic biology of SGCs, primarily due to their flattened morphology and close proximity to neurons, which complicates immunohistochemical studies and *in vivo* experiments ^7^. Additionally, SGCs rapidly change their phenotype in culture, making *in vitro* experiments similarly challenging ^25, 26^. It is therefore encouraging that recent advances in single cell RNA sequencing (scRNAseq) have made it possible to study the transcriptional profile of these cells in previously unprecedented detail. To date, six papers and preprints have included such SGC scRNAseq studies in mice, with focus on either development ^27, 28^, species comparison ^29^ or nerve injury ^10, 30, 31^. Furthermore, our team published a bulk RNA-seq experiment with focus on nerve injury ^13^.

Here, we present two additional scRNAseq datasets on mouse SGCs, which we analysed in conjunction with those previously published. Such a meta-analytic approach is especially important for scRNAseq experiments, since they are frequently performed on limited biological replicates due to financial constraints. It is therefore especially important to compare findings across studies, to investigate which changes are replicable across models and laboratories.

While we were able to identify a reproducible transcriptional nerve injury signature in SGCs, the number of genes found commonly regulated across datasets was small. Furthermore, we compared the transcriptional profiles of acutely isolated SGCs with those cultured *in vitro*. Our findings confirm that cultured SGCs indeed present a different transcriptional profile relative to those acutely isolated ^26^. Finally, we compiled the datasets we analysed and made them easily accessible at the Broad Institute’s Single Cell Portal ( https://singlecell.broadinstitute.org/single_cell/study/SCP1539/) for other scientists to investigate their genes of interest.

## Methods

### Animals

All mice were housed under standard conditions with 12h light/dark cycle and free access to standard chow and water. For the spared nerve injury (SNI) experiment, 13-week-old male C57BL/6J mice from Janvier labs were housed in pairs of 2 littermates. Small-grained bedding was used after SNI. The SNI experiment was approved by the Danish Animal Experiments Inspectorate under the Ministry of Environment and food (permission number 2017-15-0201-01192-C1). For the culture experiment, two-week-old male and female SWISS mice from Janvier labs were used. This animal experiment was conducted in accordance with the EU legislation for the care and use of laboratory animals (Directive 2010/63/EU) and the German Animal Welfare Act (“Tierschutzgesetz”, 2019).

### Spared nerve injury (SNI)

SNI was performed in the left and right hindleg according to a method described previously ^32^. The procedure was performed under isoflurane (IsoFlo Vet, Abbott) anaesthesia. The sciatic nerve was exposed with skin incision and blunt dissection of the overlying muscle. A 6.0 vicryl suture was used to tightly ligate and then cut the common peroneal and tibial branches of the sciatic nerve, with the sural nerve left intact. The wound was closed with surgical tissue adhesive (Indermil Tissue Adhesive, Henkel), and for local analgesia a droplet of lidocaine SAD (10 mg/ml; Amgros I/S) was applied to the wound. Buprenorphine (0.3 mg/ml; Temgesic, RB Pharmaceuticals) and the antibiotic ampicillin (250 mg/ml; Pentrexyl; Bristol-Myers Squibb) were mixed and diluted 1:10 in isotonic saline (9 mg/ml; Fresenius Kabi) and 0.1 ml was injected subcutaneously following surgery for peri-operative analgesia and protection against infection. The operation was performed bi-laterally to ensure enough material for the sequencing, and eight L3 and L4 DRGs were collected from 2 mice per condition (naïve, 7 days post SNI and 14 days post SNI).

### Cultured DRG cells

The 2-week-old mice were euthanized with CO _2_ before they were disinfected in 70% ethanol and decapitated. DRGs from cervical, thoracic, and lumbar levels were dissected. The ganglia were then incubated in 2.5 ml CD dissociation buffer (DMEM + GlutaMAX, Thermo Fisher, 31966-021 with 3.6 mg/ml glucose, Carl Roth, NH06.3, 3 mg/ml Collagenase type IV, Worthington, LS004186, and 6 mg/ml Dispase, Worthington, LS02109) for 40 min at 37°C. Next, the CD dissociation buffer was replaced by 5 ml D dissociation buffer (DMEM + GlutaMAX with 3.6 mg/ml glucose, and 3mg/ml Dispase) for a further 40 min at 37°C. Following enzymatic digestion, the cells were manually triturated in cell medium (DMEM + GlutaMAX with 5% horse serum, Thermo fisher, 26050-070 and 0.5% Penicillin-Streptomycin, Sigma Aldrich, P4333-20ML), after which the cellular solution was cleared of debris by gradient centrifugation through 4 layers of various percentages of OptiPrep ^TM^ (Sigma Aldrich, D1556-250ML) in cell medium (from the bottom: 28% OptiPrep with resuspended cells, 15%, 8% and 0%). The gradient was centrifuged at 800xg for 22 min, and cells were recovered from the interface between the 15% and 8% layer. The DRG cells were plated with a density of 560 neurons per mm ^2^ on laminin coated 24-well plates and kept for 72h at 37°C, 5% CO _2_.

### Dissection and processing of DRGs for scRNAseq

**SNI experiment (Cell_SNI):** Mice were anaesthetized using isoflurane and transcardially perfused using 10-20 ml DPBS (Thermo Scientific, SH3002802). L3 and L4 DRGs were identified and collected from both sides as previously described ^33^. For each time point (naïve, 7 days and 14 days post SNI) L3 and L4 DRGs were dissected from 2 mice and pooled (8 DRGs/sample) in ice-cold HBSS (Gibco, 14170088). DRGs were centrifuged for 4 min at 500 xg at 4°C and incubated in 1 ml dissociation buffer (2.5mg/ml collagenase, Sigma Aldrich, C9722, and 5 U/ml dispase II, Sigma Aldrich, D4693 in DPBS) for 30 min at 37°C in 5% CO _2_. Following enzymatic digestion, the cells were manually triturated using a p1000 pipette until homogenous. 9 ml of PBS was added (Sigma, D8537), and cells were centrifuged at 500xg for 8 min, 4°C and incubated for 10 min at 37°C in 0.5ml trypsin-EDTA (0.25% trypsin w/v and 0.1% EDTA w/v, Sigma 59418C diluted 1:1 in DPBS). 5 ml HBSS with 10% (v/v) Fetal Bovine Serum (FBS, Sigma, F9665) was added to stop the reaction. The cell suspension was centrifuged at 500xg, 4°C for 8 min, and the cell pellet resuspended in 1 ml HBSS with 40 Kunitz units Deoxyribonuclease I (Sigma Aldrich, DN25-1G) before filtration through a 40µm cell strainer (VWR, 734-0002). Following a final centrifugation for 10 min at 500 xg, 4°C the cells were resuspend in PBS, 5% (v/v) FBS at a concentration of 1000 cells/ul.

**Cultured DRG cells (Cell_culture):** The cells were maintained in culture for 72h before they were detached with Trypsin-EDTA 0.25% w/v, centrifuged, counted, and processed for 10X scRNAseq.

### scRNAseq on 10X Chromium (Cell_SNI and Cell_Culture)

To construct scRNAseq libraries, the cell suspensions were processed with the Chromium Single Cell 3’ GEM, Library & Gel Bead Kit v3 (10x Genomics, PN 1000075) according to the manufacturer’s instructions. During this process, the 10X Chromium device uses a microfluidic system to partition each cell into a single droplet, each containing sequencing barcodes and the enzymes required for reverse transcription. The barcodes are specific to each droplet and ensure that it is possible to identify which transcripts were detected in which cell after sequencing. The libraries were sequenced using DNBSEQ-G400. The raw sequencing reads were processed using Cell Ranger version 3.0.2 and mapped to the reference genome mm10-3.0.0, Ensemble 93 (see Table 1 for cell numbers and mapping statistics). The three Cell_SNI conditions (naïve, 7 days, 14 days) were processed on the same 10X chip and in the same subsequent library preparation, in order to minimise batch effects. Equally, the two replicates of the cultured cells were processed on the same 10X chip.

**Table 1.** Meta-data of the Cell_SNI and Cell_culture datasets.

	Estimated cell number	Mean reads per cell	Median genes per cell
**Cell_SNI, naïve**	3,748	111,532	1,508
**Cell_SNI, 7 days**	3,281	121,501	1,824
**Cell_SNI, 14 days**	4,908	84,366	1,856
**Cell_culture, A**	5,175	14,948	2,366
**Cell_culture, B**	5,773	12,764	2,210

### Quality control of scRNAseq

The Cell_SNI and Cell_culture count matrices were analysed with Seurat v3 ^34^ in R. The previously published datasets with focus on nerve injuries ^10, 30, 31^ were reanalysed with Seurat v3 from the count matrices made available on the Gene Expression Omnibus website (GSE139103, GSE154659 and GSE155622). To ensure that we analyse high quality cells, we started by filtering out those with less than 200 detected genes. We further filtered out likely dead cells, based on mitochondrial gene expression permitting a maximum of 30% gene expression being mitochondrial, tailoring the precise cut-off value to each individual dataset ( Table 2).

**Table 2.** Overview of details for analysis of the datasets. All cells passed the quality control in the Renthal *et al.* and Wang *et al.* datasets because the used datasets had already been through pre-processing.

Dataset	Cut-off for mitochondrial genes (%)	Number of cells after quality control	Resolution for clustering	Dimensions (PCA and clustering)	Doublet cut-off for SGC cluster (# nFeature_RNA)
**Cell_SNI**	30%	10901 out of 11937	0.08	1:20	3500
**Cell_crush**	15%	6838 out of 6975	0.08	1:20	2500
**Cell_culture**	10%	10563 out of 10948	0.18	1:20	NA
**Renthal *et al.***	10%	141093 out of 141093	0.5	1:20	NA
**Wang *et al.***	5%	20806 out of 20806	0.08	1:20	NA

### Clustering and visualization of scRNAseq

Next, we performed normalization of the raw transcript counts detected in each cell. The normalization is a two-step process consisting of scaling and transformation. Scaling is performed by calculating counts per 10,000 counts. This provides count concentration instead of absolute number, which is useful since cells vary in size and therefore also in number of mRNA molecules. Furthermore, scaling removes efficiency noise, which arises because the v3 chemistry used for sequencing is not equally effective in each droplet. Next, natural-log transformation using log1p is performed on each scaled count number for each gene in each cell. This ensures that highly expressed genes are not given more weight in the downstream integration analysis compared to lowly expressed genes.

Following the standard Seurat pipeline for clustering and integration of datasets, we next identified the 2000 genes that showed the highest cell-to-cell variability using the parameter “vst” with the FindVariableFeatures function. The genes with the highest cell-to-cell variability can also be determined by other methods such as “mean.var.plot” or “dispersion”. Using all three methods we found gene lists and showed that the subsequent cell clustering is stable with the different methods. We used the “vst” method as our primary method of identifying the most variable genes throughout the remaining analyses. For information on the mathematical framework for identification of highest variable genes please consult the Seurat documentation. Where necessary (i.e. when comparing across 10X chips/experiments from different laboratories), we used these genes to integrate the datasets. Integration mitigates the impact of batch effects on subsequent cluster analysis ^35^.

After variable gene identification, we applied a linear transformation step and performed PCA, which is used as the foundation for clustering and Uniform Manifold Approximation and Projection (UMAP, Table 2). UMAP provides a two-dimensional reduction, enabling visualization of the datasets, while the clustering identifies similar cells. Finally, by comparing all genes expressed in each cluster, we identified the genes that are highly expressed (marker genes) and used those to annotate the clusters with a cell type. Full R analysis scripts are available in the supplementary (Extended notebook).

### Comparison of dataset annotations with scMAP

The annotations of the clusters in the Cell_crush dataset from Avraham *et al.* ^10^ and the Cell_SNI dataset were compared to each other with scMAP ^36^ to ensure annotation consistency. We used scMAP to project each cell in the Cell_SNI dataset to the cell types identified in the Cell_crush dataset. The projection is based on the 500 most informative genes identified with the selectFeatures function in the scMAP package (Extended notebook). The selectFeatures function uses a linear model to capture the relationship between mean expression and number of dropouts (zero expression). The most informative genes are identified as the ones with more dropouts than expected, i.e. those not present in some clusters. The output of the scMAP projection is a Sankey plot, illustrating how the annotations in the datasets compare to each other.

### Additional quality control of the SGC cluster

With droplet-based sequencing technologies like 10X, there is a risk of doublets, with two cells being captured in the same droplet, and barcoded as one. An often-used strategy to eliminate doublets is to set a threshold for the number of detected genes in each cell. However, the cell types contained within a DRG are very heterogenous, ranging from very large sensory neurons to small immune cells. The difference in cell size results in the detection of relatively many genes in neurons and fewer genes in the immune cells (see Extended Figure 1) ^37^. Due to this heterogeneity, it is not possible to set a universal threshold that filters out SGC doublets without depleting neurons. Since all our downstream analyses focused on SGCs only, we subset the SGC cluster and adjusted our duplet-filtration threshold to fit this particular cell population (Extended notebook and Table 2). 

### Differentially expressed genes in SGCs

Differentially expressed genes were identified with Seurat v3 based on the unintegrated data (including all genes) using the non-parametric Wilcoxon rank sum test. For us to consider a gene to be differentially expressed, it needed to be present in at least 10% of SGCs in either the naïve or injured conditions, have a log2 fold change of at least 0.25 and an adjusted p value of less than 0.05. To avoid introducing technical artefacts, we only performed these analyses within individual batch-controlled datasets (Cell_SNI and Cell_crush; i.e. those deriving from the same 10X chip) and then compared the resulting lists of differentially expressed genes across studies. The gene ontology enrichment was done with Metascape ^38^ using their web interface for multiple lists. 

### Comparison of isolated and cultured SGCs (Cell_SNI versus Cell_culture)

The Cell_SNI and Cell_culture datasets were integrated using Seurat v3. Joint clusters were identified and annotated as described above. To investigate the SGC cluster further, we subset it and performed normalization, integration, clustering and visualization again on the raw counts. This resulted in 5 different SGC subclusters. We compared the transcriptome of our joint SGC dataset to a scRNAseq dataset of the developing mouse nervous system from Furlan *et al.* ^39^, using the matchReferences() function of SingleR ^40^ (see Extended notebook for more details). The function finds the probability of a cell in the SGC dataset being assigned each label in the dataset from Furlan *et al.* and vice versa. A probability of 1 indicates that there is a 1:1 relation between that pair of labels while a probability of 0 indicates that the cell clusters are not similar.

## Results

### Cell annotations in different scRNAseq data sets

In this analysis four different sets of single cell or single nucleus RNAseq data from mouse DRGs after different nerve injuries (see Table 3) were included. Three datasets are published ^10, 30, 31^ and available online (GSE139103, GSE154659 and GSE155622) while the fourth scRNAseq dataset of SNI responses at day 7 and 14 in the DRG is published in this work (GSE174430). The overall goal of this analysis was to identify if SGCs share a common response to nerve injuries across different experimental conditions.

**Table 3.** Overview of the different single cell and single nucleus RNAseq datasets analysed in this paper.

Publication	Dataset	Mouse strain	Age	Sample prep	Condition	scRNA-seq	Included in injury response analysis	Included in culture comparison analysis
**Avraham** ***et al.* 2020**	**Cell_crush**	**C57Bl/6J**	**8-12 weeks**	**Dissociation** **with collagenase** **and papain,** **sorted with FACS**	**Crush, 3** **days**	**Whole cell, 10X**	**Yes**	**No**
**N/A**	**Cell_SNI**	**C57Bl/6J**	**13 weeks**	**Dissociation** **with collagenase** **and dispase,** **followed by** **trypsin**	**SNI, 7 &** **14 days**	**Whole cell, 10X**	**Yes**	**Yes**
**N/A**	**Cell_culture**	**SWISS**	**2 weeks**	**Dissociation** **with collagenase** **and dispase**	**uninjured,** **cultured** **for 3 days**	**Whole cell, 10X**	**No**	**Yes**
Renthal *et al.* 2020	N/A	C57Bl/6J	8-12 weeks	Extraction of nuclei	various injuries	Nucleus, InDrops	No	No
Wang *et al.* 2021	N/A	C57Bl/6J	7-8 weeks	Dissociation with enzymes, sorted with Percoll gradient	SNI at various time points	Whole cell, 10X	No	No

First, the published datasets were re-analysed, focusing specifically on SGC clusters. It was apparent that the SGC scRNAseq data from Renthal *et al.* and Wang *et al.* contain a substantial amount of neuronal background signal. Specifically, the top differentially expressed genes after nerve injury in non-neuronal cell types all resemble the same ‘canonical’ neuronal response profile. For example, genes such as Gal, Atf3, Npy, Nts and Sprr1a were regulated in the SGC clusters (see Extended Figure 2 and Extended Excel Sheets “Renthal et al 7d” and “Wang et al 7d” for the full list of differentially expressed genes). While both studies contain an impressive amount of data, with cells taken from many different time points and nerve injury models, both were also designed with a focus on DRG neurons – and as it turns out, this impacts their suitability for the analysis of differential gene expression in non-neuronal cells. To avoid any bias in our SGC analysis, these datasets therefore had to be excluded from the meta-analysis.

**Figure 1.**
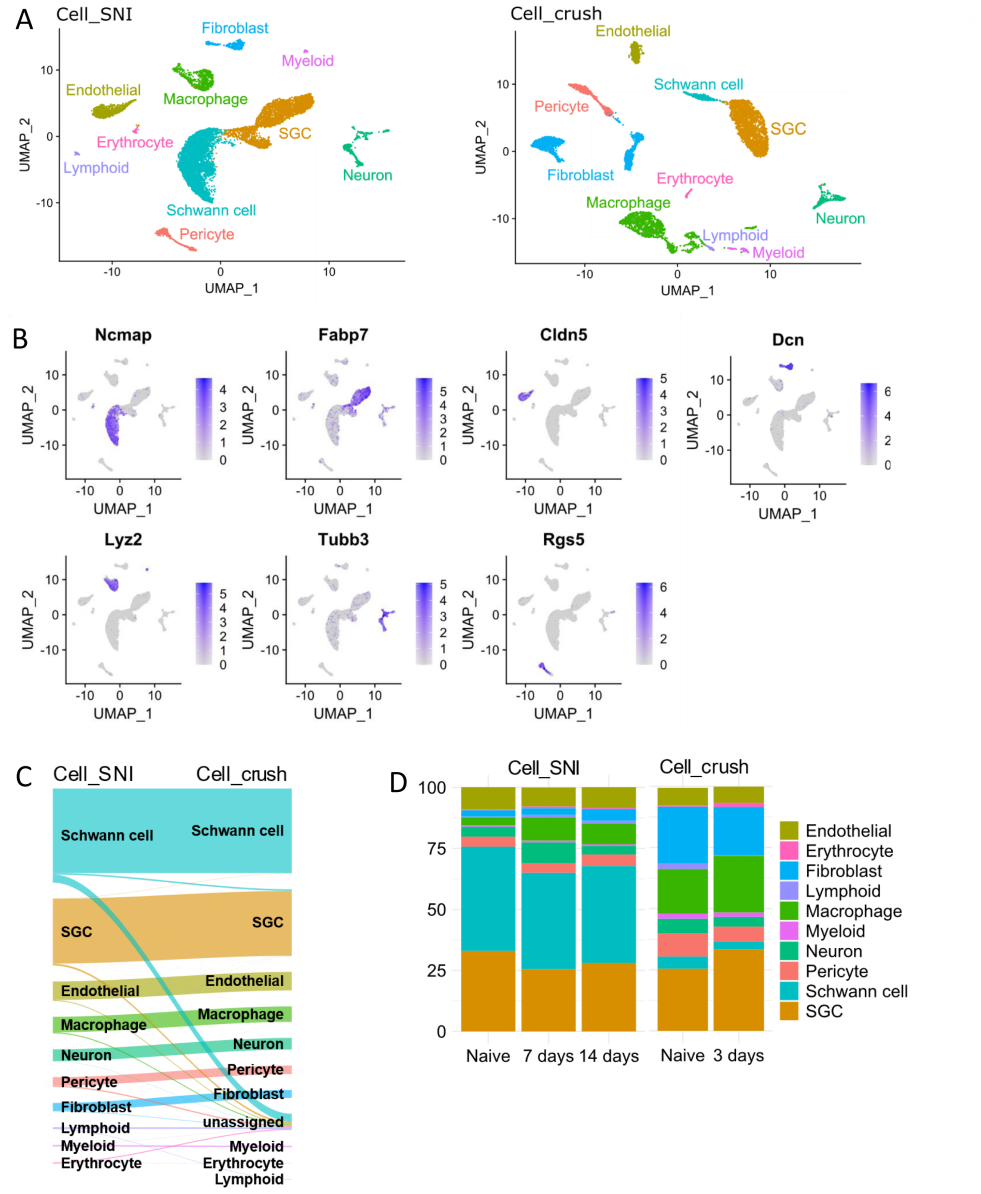
SGCs are easily identifiable in the Cell_SNI and Cell_crush datasets. **A**) UMAPs of the Cell_SNI and Cell_crush datasets highlighting the identified cell types. **B**) UMAPs of the Cell_SNI dataset highlighting gene expression used to identify the cell types. Ncmap = Schwann cells, Fabp7 = SGCs, Cldn5 = endothelials, Dcn = fibroblasts, Lyz2 = macrophages, Tubb3 = neurons, Rgs5 = pericytes. **C**) Sanky diagram showing the projection of the Cell_SNI dataset on to the Cell_crush dataset. **D**) The percentage distribution of the cell types in the dataset. Cell_SNI naïve = 3486 cells, Cell_SNI 7 days = 3029 cells, Cell_SNI 14 days = 4386 cells, Cell_crush naïve = 3090 cells, Cell_crush 3 days = 3748 cells

From the two remaining datasets, the sciatic nerve crush data from Avraham *et al.* (Cell_crush) ^10^ and the sciatic nerve ligation dataset (Cell_SNI) presented here, different cell populations were identified using unsupervised clustering and visualised with UMAP plots ( Figure 1A). To determine the nature of each cell cluster, the expression of marker genes was investigated ( Figure 1B) and the datasets were annotated individually based on the top marker genes for each cell type ( Figure 1A). The annotations were shown to be consistent between datasets using the package scMAP, which projects one dataset annotation on to the other ( Figure 1C). No differences in cell types present in the datasets were detected, however, minor variations in the cell proportions were observed. Specifically, more Schwann cells were detected in Cell_SNI, and more fibroblasts and macrophages in Cell_crush ( Figure 1D). This phenomenon is presumably due to the different dissociation techniques applied ( Table 3).

### SGCs demonstrate a common response to nerve injury across tested conditions and timepoints

The response of SGCs to nerve injury was investigated. Both datasets contain SGCs from L3-L5 DRGs following sciatic nerve injury. Many, but not all, neuronal somata in these DRGs project their axons to the sciatic nerve ^41^. Consequently, not all SGCs in the samples from injured conditions would have been surrounding an injured neuron.

**Figure 2.**
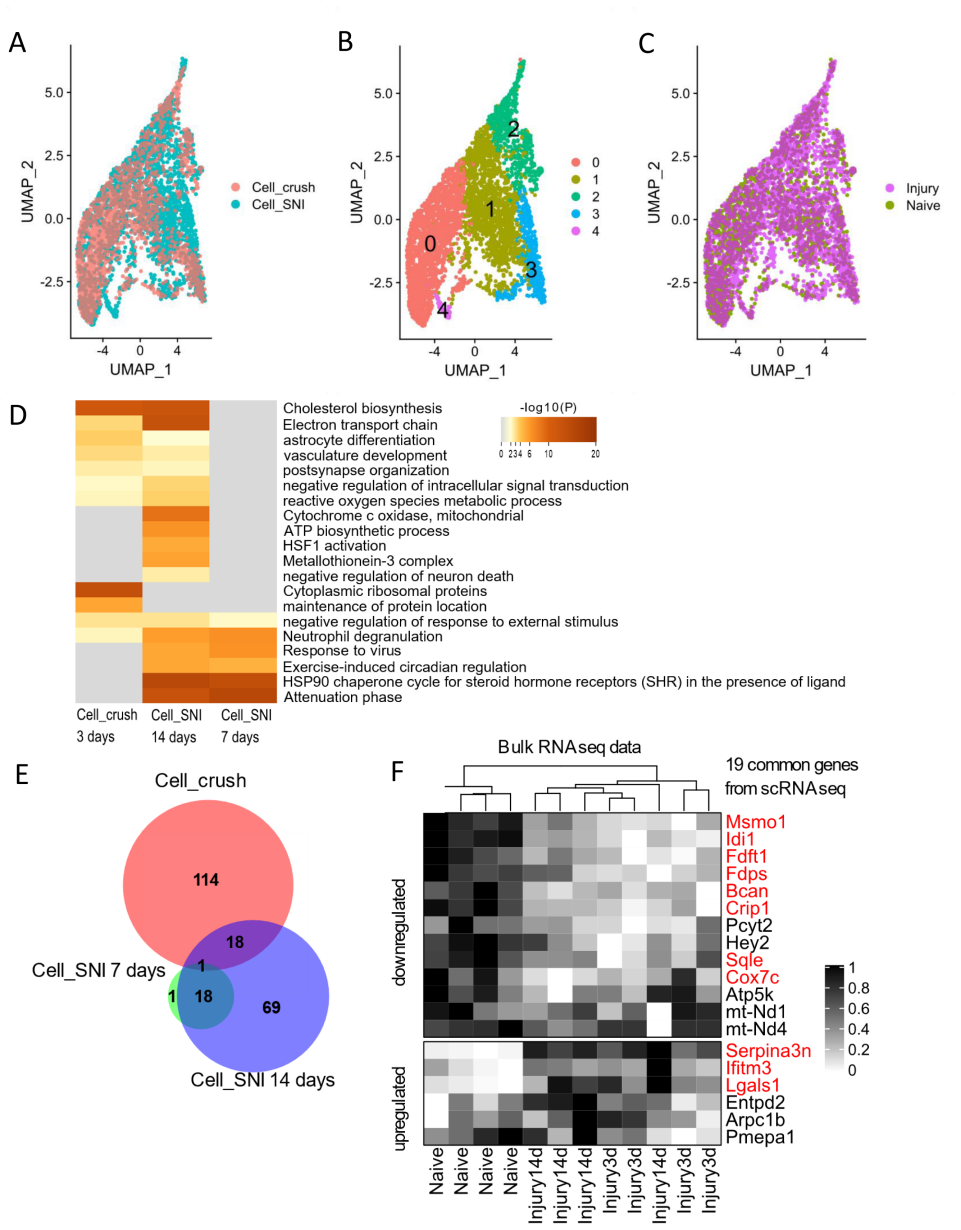
Common injury response of SGCs. **A**– **C**) UMAPs of combined and integrated SGCs from the Cell_SNI (3153 cells) and Cell_crush (2147 cells) datasets. **A**) UMAP coloured based on dataset. **B**) UMAP coloured based on clustering. Each number/colour denotes a cluster. **C**) UMAP coloured based on injury condition with 3321 SGCs from injured condition and 1979 SGCs from uninjured condition. **D**) Heatmap of enriched gene annotation terms. The top 20 highest ranking terms are shown. **E**) Venn diagram of number of differentially expressed genes in SGCs in Cell_crush and Cell_SNI datasets when comparing injured states to naïve. **F**) Heatmap displaying expression levels from bulk RNAseq data (Jager et al) containing n=4 for per condition (naïve, 3 days and 14 days after injury). The genes extracted here are the 18+1 common genes between the Cell_SNI and Cell_crush datasets from Figure 2C. The genes marked in red are also differentially regulated in the displayed bulk RNAseq.

First, it was assessed if unsupervised cluster analysis could distinguish SGCs that had been surrounding an injured neuron from those that had not. To ensure that the analysis contained enough data to enable sub-clustering, the SGCs from the two datasets were combined and integrated ( Figure 2A). The integration and cluster analysis were performed using the 2000 most variable genes expressed in the SGCs using the “vst” method (see Methods). It was, however, not possible to identify a cluster consisting exclusively of SGCs from injured mice, neither when analysing all SGCs integrated together ( Figure 2B and C, Extended table 1), nor when analysing them individually within each dataset (Extended Figure 3). The analysis was repeated with alternative methods (“dispersion” or “mean.var.plot”) of identifying the 2000 variable genes (Extended figure 4). Even though the lists of genes with the highest cell-to-cell variability differs between the 3 different methods of analysis, the subsequent cell clustering did not identify clusters containing only SGCs from the injured condition (Extended figure 5, Extended table 1). This suggests that the differences induced by nerve injury are comparatively more subtle in SGCs than in DRG neurons, which clearly cluster together when damaged ^30^. It also suggests that, if an injury-specific SGC cluster were to exist, it would require substantially larger datasets, with more cells increasing the chance of detecting more subtle changes in the SGC transcriptome.

Next, differentially expressed genes were identified by comparing all SGCs from the injured sample with those from the naive. The differential analysis was performed within each dataset to avoid adding batch effects and additional noise. In the Cell_SNI dataset, SGCs from seven days (794 cells) and 14 days (1268 cells) after nerve injury were compared to the gene expression in SGCs from naïve mice (1210 cells). In the Cell_crush dataset SGCs from three days (1318 cells) after a crush injury were compared to SGCs from naïve mice (829). 

Despite differences in both time point and injury type, common differentially regulated genes were found to be enriched in related gene annotation groups ( Figure 2D and Extended Excel Sheet: DE_analysis_metascape). For example, in both Cell_SNI at 14 days and Cell_crush at three days, genes were enriched in cholesterol biosynthesis (Extended Figure 6 and Extended Excel Sheet: DE_analysis_metascape).

To identify which specific regulated genes the Cell_SNI and Cell_crush datasets have in common, the lists of differentially expressed genes were compared ( Figure 2E). 18 genes were identified as common between Cell_SNI at 14 days and Cell_crush at three days – an enrichment that is 12x larger than expected by chance (as determined by hypergeometric probability calculations, assuming a total population of 10,000 genes as being expressed in SGCs). The common genes include five genes of the cholesterol biosynthesis pathway which contains a total of 15 genes (WP103 from WikiPathways). The five commonly regulated cholesterol biosynthesis genes are: Idi1, Msmo1, Fdps, Fdft1 and Sqle ( Table 3). These genes were not detected as regulated at seven days after SNI (Extended Figure 6 and Extended Excel Sheet: DE_analysis_metascape).

We have previously performed bulk RNAseq on sorted SGCs three and 14 days after transection of the sciatic nerve ^13^. To check whether scRNAseq and bulk RNAseq are in agreement, the 19 common genes identified between the Cell_SNI and Cell_crush datasets were compared to the gene expression in the bulk dataset ( Figure 2F). Of these 19 genes, 11 genes are also significantly regulated in the bulk dataset and in the same up/down direction ( Figure 2F and Table 4), confirming the regulation of the genes involved in cholesterol biosynthesis (Idi1, Msmo1, Fdps, Fdft1 and Sqle).

**Table 4.** List of the 19 common regulated genes including log2 fold change and general gene function.

Gene	Cell_crush 3 days	Cell_SNI 7 days	Cell_SNI at 14 days	Bulk 3 days	Bulk 14 days	Gene function
**Arpc1b**	0.80	#N/A	0.27	#N/A	#N/A	Involved in DNA damage
**Lgals1**	0.84	#N/A	0.34	1.65	1.96	Regulating apoptosis
**mt-Nd1**	-0.30	#N/A	0.27	#N/A	#N/A	Mitochondrial
**mt-Nd4**	-0.30	#N/A	0.32	#N/A	#N/A	Mitochondrial
**Bcan**	-0.53	#N/A	-0.38	-0.70	-0.69	Extracellular matrix
**Msmo1**	-0.42	#N/A	-0.39	-1.03	-0.78	Sterol metabolic process
**Cox7c**	-0.28	#N/A	-0.27	#N/A	-0.41	Mitochondrial
**Serpina3n**	0.58	#N/A	0.66	2.33	2.56	Inhibits proteases
**Fdps**	-0.39	#N/A	-0.33	-0.82	#N/A	Sterol metabolic process, Cholesterol biosynthesis
**Idi1**	-0.43	#N/A	-0.42	-1.32	#N/A	Sterol metabolic process, Cholesterol biosynthesis
**Crip1**	-0.41	#N/A	-0.27	-0.70	-0.66	AT DNA binding
**Entpd2**	0.52	#N/A	0.33	#N/A	#N/A	Nucleoside-diphosphatase activity
**Sqle**	-0.35	#N/A	-0.27	-0.64	-0.47	Sterol metabolic process
**Hey2**	-0.29	#N/A	-0.27	#N/A	#N/A	Transcription factor
**Atp5k**	-0.25	#N/A	-0.29	#N/A	#N/A	Mitochondrial
**Pcyt2**	-0.25	#N/A	-0.26	#N/A	#N/A	Phospholipid synthesis
**Fdft1**	-0.26	#N/A	-0.30	-0.84	-0.65	Sterol metabolic process, Cholesterol biosynthesis
**Pmepa1**	-0.27	#N/A	-0.31	#N/A	#N/A	Negative regulation of TFGbeta signaling
**Ifitm3**	0.42	-0.5654	#N/A	#N/A	0.69	Interferon induced membrane protein

### Regulation of known SGC markers

The list of common regulated genes ( Table 3) includes several that have yet to be investigated in the context of SGC function. Surprisingly, the list did not include genes that have previously been reported to be regulated at protein or gene level such as Connexin43 ( *Gja1*), GFAP ( *Gfap*) or Hmgcs1 ( *Hmgcs1*) ^42– 44^. Therefore, these genes were further examined in the datasets (2x scRNAseq, 1x bulk RNAseq). Connexin43 has been shown to be increased at protein level in SGCs after nerve injury ^42, 43^. Counterintuitively, a downregulation of *Gja1* at the mRNA level in the bulk RNAseq were observed while no regulation of *Gja1* in the scRNAseq datasets were detected ( Table 5).

**Table 5.** Analysis of differential expression of Gja1, Gfap and Hmgcs1 in SGCs in various datasets. % = % of SGCs expressing Gja1 (gene for Connexin43), Gfap and Hmgcs1. FPKM = Fragment per kilobase of transcript per million mapped reads.

Dataset and time point	%	FPKM	Log2 foldchange	Adj P-value
Gja1				
Cell_SNI 7 days	47	N/A	-0.05	N/A
Cell_SNI 14 days	40	N/A	-0.24	N/A
Cell_crush 3 days	31	N/A	-0.20	N/A
Bulk 3 days	N/A	88	-0.58	0.02
Bulk 14 days	N/A	100	-0.46	0.001
Gfap				
Cell_SNI 7 days	3	N/A	0.1	N/A
Cell_SNI 14 days	7	N/A	0.24	N/A
Cell_crush 3 days	3.7	N/A	0.26	N/A
Bulk 3 days	N/A	0.33	1.8	N/A
Bulk 14 days	N/A	0.41	2.2	N/A
Hmgcs1				
Cell_SNI 7 days	71	N/A	-0.1	N/A
Cell_SNI 14 days	72	N/A	-0.24	N/A
Cell_crush 3 days	66	N/A	-0.43	1.2 * 10 ^-29^
Bulk 3 days	N/A	217	-0.93	1.9*10 ^-14^
Bulk 14 days	N/A	269	-0.69	4*10 ^-9^

Increased expression of GFAP protein is often used as a marker for SGC reactivity by immunohistochemical analysis ^42, 43, 45, 46^. In bulk RNAseq, *Gfap* gene expression was not detected above threshold (FPKM>1), as we previously described ^13^. In accordance with this, expression of *Gfap* in the scRNAseq datasets (Cell_SNI and Cell_crush) were only detected in 3 – 7% of SGCs, which is below our defined threshold (see methods). Furthermore, we did not observe differential regulation of *Gfap* above threshold in either dataset ( Table 5). Whether this result reflects strain or species variation is discussed elsewhere ^47^.

Finally, the cholesterol synthesis pathway enzyme Hmgcs1 has been shown to be downregulated in SGCs after nerve injury ^44^. In our datasets, significant transcriptional downregulation of *Hmgcs1* in the Cell_crush dataset and the bulk RNAseq after sciatic nerve ligation ( Table 5) was detected. We did not confirm *Hmgcs1* downregulation in the Cell_SNI dataset, however downregulation of other genes involved in the biosynthesis of cholesterol were observed, supporting injury-induced regulation of the cholesterol synthesis pathway in SGCs (Extended Figure 6 and Extended Excel Sheet: DE_analysis_metascape).

### Transcriptional response in cultured glia cells

**Figure 3.**
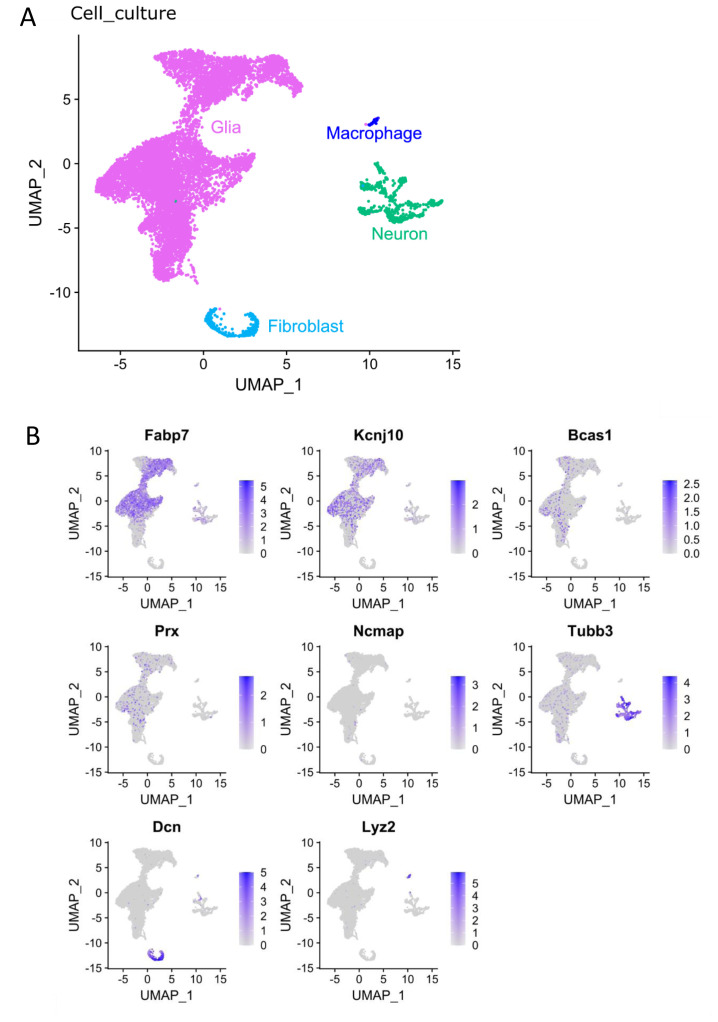
The Cell_culture dataset contains glial cells, fibroblasts, neurons, and macrophages. **A**) UMAP of the identified cell types in the Cell_culture dataset. 9332 glial cells, 764 neurons, 385 fibroblasts and 82 macrophages. **B**) Expression of markers for SGCs (Fabp7 and Kcnj10), Schwann cells (Bcas1, Prx, Ncmap), neurons (Tubb3), fibroblasts (Dcn) and macrophages (Lyz2) in the Cell_culture dataset.

SGCs have on several occasions been investigated using *in vitro* cultures from either pups or adult rodents ^48– 51^. However, reports of loss of marker protein expression upon disconnection from their associated neuron ^25^ as well as regression to a transcriptional profile expressing 99.8% of the same genes as cultured Schwann cells ^26^ complicate meaningful translational interpretations to the *in vivo* condition. Here scRNAseq was performed on primary cultures of mouse DRGs (Cell_culture, GSE188971) to compare the transcriptional profiles of such cultured SGCs to that of acutely isolated naïve and injured SGCs of the Cell_SNI dataset. When performing the initial cluster analysis of the Cell_culture dataset, 4 different clusters of cells were identified: neurons, macrophages, fibroblasts and glial cells ( Figure 3A), with glial cells constituting the vast majority (88%). The glia cell cluster was explored in the attempt to subdivide further by relying merely on the expression of traditional Schwann cell and SGC markers ( Figure 3B). However, the SGC markers *Fabp7* and *Kcnj10* (Kir4.1) showed no clear SGC clustering, and Schwann cell markers were even more widely dispersed, indicating that glial cells change their gene expression profiles extensively *in vitro*.

**Figure 4.**
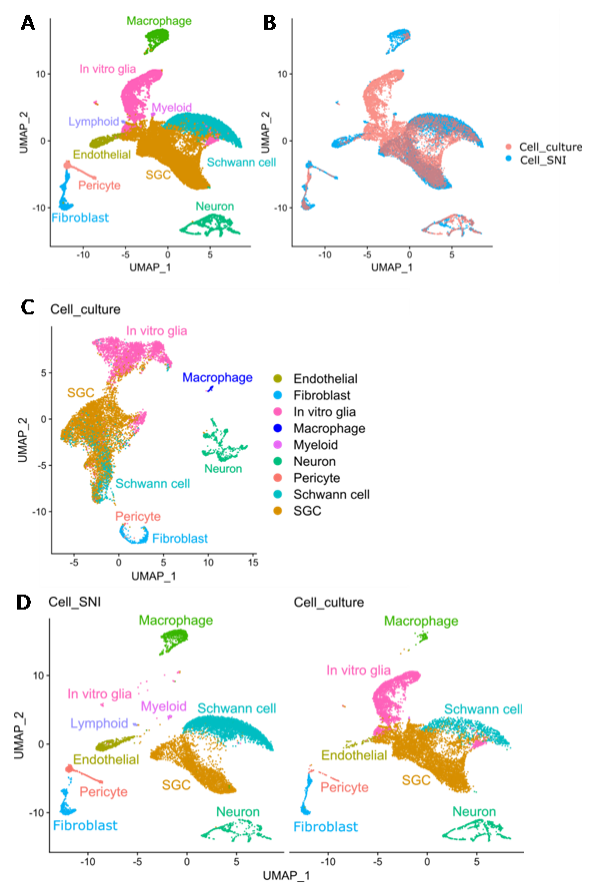
Glia cells change in culture. **A**) UMAP of joint analysis of the Cell_culture (10563 cells) and Cell_SNI (10901 cells) datasets with annotation of cell types. **B**) UMAP of joint analysis of the Cell_culture and Cell_SNI datasets coloured based on dataset. **C**) UMAP of Cell_culture dataset with annotation from joint analysis. **D**) UMAPs of the joint analysis split based on dataset origin (Cell_culture or Cell_SNI) with annotation of cell types identified from the joint analysis. **E**) Expression of markers for SGCs (Fabp7 and Kcnj10), cell proliferation (Top2a and Mki67) and Schwann cells (Ncmap, Bcas1 and Prx) in the joint analysis. **F**) Heat map showing the result of the SingleR analysis which compared the gene expression in the joint analysis (Cell_culture and Cell_SNI datasets) with cells in the developing peripheral nervous system. SCP = Schwann cell precursor.

To improve annotations and investigate translational variations of cultured SGCs relative to their *in vivo* state, the Cell_culture dataset was integrated with the Cell_SNI dataset to enable joint analyses ( Figures 4A and 4B). Cell culture glia cells clustered together with acutely isolated SGCs and Schwann cells ( Figure 4B). A projection of the integrated annotation back onto the Cell_culture dataset pre-integration is illustrated in Figure 4C and shows that the glia cluster ( Figure 3A) indeed contains many different cell types. The joint analysis also reveals a distinct glial cell cluster (“ *In vitro* glia”), selectively present in the Cell_culture dataset ( Figure 4D). The “ *In vitro* glia” cluster is also identified when performing the cluster analysis with the 2000 most variable genes identified with “mean.var.plot” or “dispersion” (see Extended table 2). The “ *In vitro* glia” cells are enriched for SGC marker genes such as *Fabp7* and *Kcnj10*, and for genes involved in cell proliferation, such as *Top2a* (DNA topoisomerase II alpha) and *Mki67* (marker of proliferation Ki-67), but do not express the Schwann cell markers *Ncmap*, *Bcas1* or *Prx* ( Figure 4E). It has previously been suggested by George *et al.* that peripheral glia cells regress back to a Schwann cell precursor (SCP) phenotype when cultured ^26^. To investigate if this could be the fate of the “ *in vitro* glia” cluster, the joint dataset was compared to a dataset containing cells from the developing peripheral nervous system at E12.5 ^39^ with the R package SingleR ^40^. Besides SCPs, the developing nervous system includes neuroendocrine chromaffin cells, bridge cells (which are an intermediate fate-state between SCPs and chromaffin cells), and sympathoblasts that develop into sympathetic nerve cells or chromaffin cells ^39^. The analysis shows that the “ *in vitro* glia” cluster does indeed resemble SCPs ( Figure 4F), supporting the hypothesis that peripheral glia regress into a SCP phenotype in culture. As expected, the neuronal cluster has similarities with sympathoblasts ( Figure 4F).

### SGCs change toward a precursor phenotype *in vitro*

**Figure 5.**
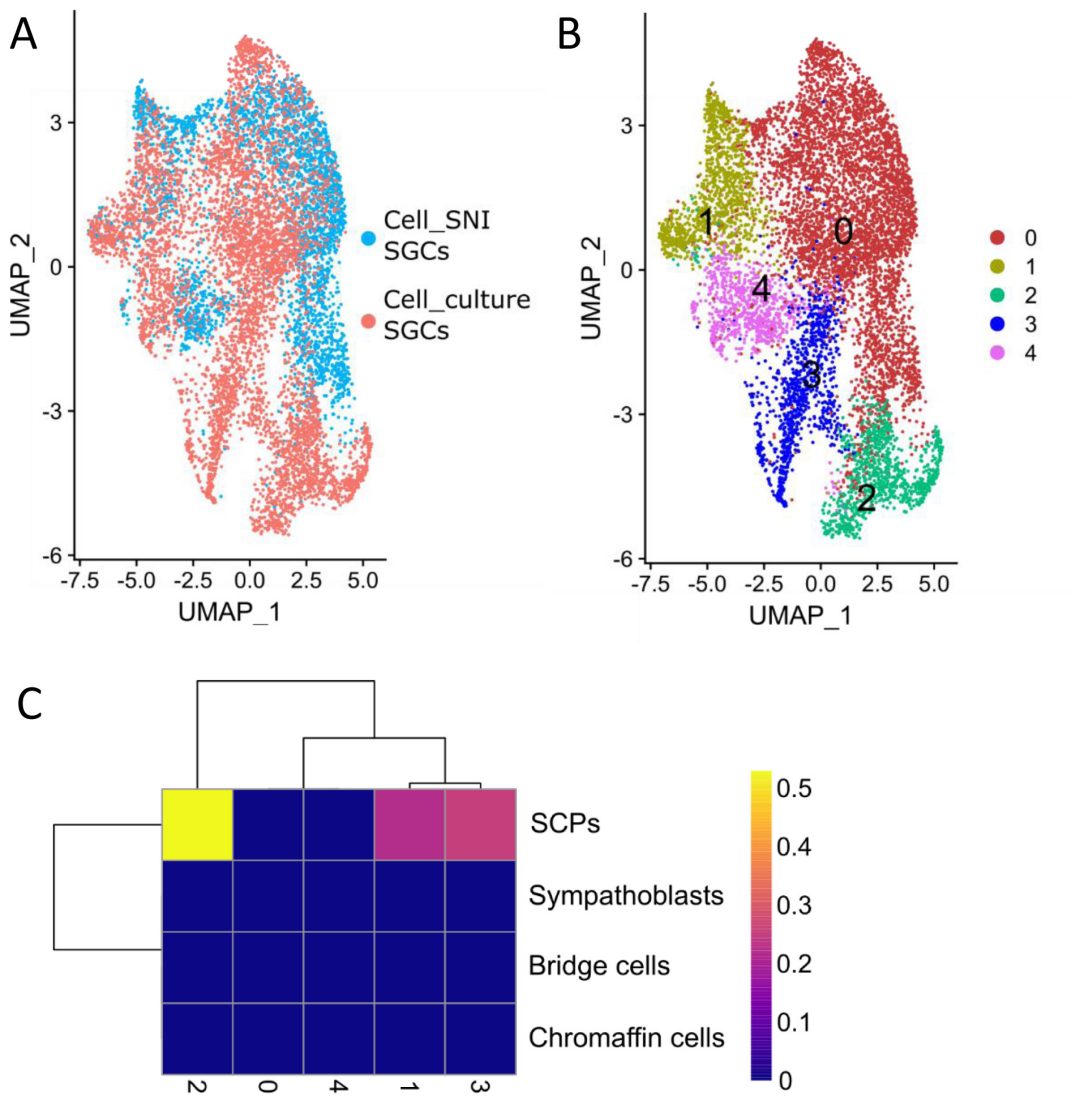
Some SGC change towards a precursor phenotype in vitro. **A**) UMAP of the 8783 SGC from the joint analysis of the Cell_culture and Cell_SNI dataset coloured by dataset. **B**) UMAP of SGC cluster from joint analysis of the Cell_culture and Cell_SNI dataset with cluster analysis performed only on the SGCs. **C**) Heat map showing the result of the SingleR analysis which compared the gene expression in the SGC clusters with cells in the developing peripheral nervous system. SCP = Schwann cell precursor.

Finally, the joint analysis was used to identify differences between the SGCs originating from the Cell_culture or the Cell_SNI dataset (orange SGC cluster in Figure 4A). To increase the resolution for the SGC cluster, it was subset and re-clustered. This showed that a significant number of the cells from the Cell_culture condition cluster separately ( Figure 5A and B), suggesting that their transcriptional profile diverges significantly from those of Cell_SNI SGCs. This was particularly the case for cells in subclusters 2 and 3 ( Figure 5B).

To investigate whether these culture-induced changes also point to a regression towards a SCP phenotype, the 5 SGC clusters were compared to the cell types in the developing nervous system ^39^ with SingleR ^40^. The results revealed that cluster 2 resemble SCPs ( Figure 5C), raising the possibility that, in addition to “ *in vitro* glia”, a proportion of SGCs in culture revert to a mutual precursor phenotype.

## Discussion

In the last few years, single nucleus and scRNAseq datasets have been published to investigate the injury response of DRG cells, with a particular focus on neurons ^30, 31^ and SGCs ^10^. With this study, we adopted a meta-scientific approach to summarise specifically the over-arching conclusions that can be drawn from these data on how SGCs behave after nerve injury. From the 4 datasets we considered for inclusion, two ^30, 31^ were excluded due to high levels of neuronal contamination in the differential expression analysis. The reasons for this contamination are not clear. As all the investigated non-neuronal cell types and not only the SGCs have the ‘canonical’ neuronal response, we find it unlikely that it should be due to insufficient disruption of the SGC-neuron units. Instead, we believe that it may be related to the magnitude of transcriptional regulation in neurons, which dwarfs that of all other DRG cell types following nerve injury. This greater response can be a source of cross-contamination if neuronal mRNA is present in the cellular mixture before droplet separation. In the case of Renthal *et al.*, significant amounts of cytosolic mRNA would have been released during the isolation of nuclei just prior to their single-nucleus RNAseq. In the case of Wang *et al.*, their neuronal enrichment step result in more neurons being sequenced than in the SGC-focused datasets. We speculate that this would also have been accompanied by a proportional increase in the number of dead neurons (i.e. free neuronal mRNA) in the starting cell mixture.

Analysing the two remaining datasets, we identified a common SGCs transcriptional injury response, with downregulation of genes annotated to cholesterol biosynthesis. This finding is in line with protein data published by Wang *et al.* ^44^, who reported downregulation of the cholesterol pathway protein Hmgcs1 in rat DRG after spinal nerve ligation. Little is known about the possible functional consequences of this potential change in cholesterol metabolism. After nerve injury, it has been shown that SGCs increase their cell membrane surface area ^23^. It seems counter-intuitive that there can both be a downregulation of cholesterol production and an increased membrane production, considering mammalian plasma membranes consist of approx. 30% cholesterol ^52^. One might wonder whether SGCs change how they obtain their cholesterol after nerve injury. Since they express general cholesterol receptors, like LDLR and VLDLR, they would be capable of taking up cholesterol from the extracellular space, where it might be released from activated macrophages. Macrophages are known for their high cholesterol production, and we and others have shown that they increase in number and migrate into the SGC-neuron unit after injury ^13, 53– 56^. At present, however, this remains speculation until more functional data can be obtained.

When performing sciatic nerve injuries on mice, not all neurons in the corresponding DRG (L3-L5) will be injured ^41^. Consequently, we expect not all SGCs in the injured samples to have an injury response. We were therefore surprised to see that SGCs did not cluster in two groups based on whether they surrounded injured neurons or not. We speculate that the transcriptional response is too subtle to allow for sub-clustering of the SGCs into injured and uninjured cells, at least amongst the transcripts we were able to capture with droplet-based methods and the 4581 SGCs analysed here (2016 SGCs from Cell_crush and 2565 SGCs from Cell_SNI).

Beyond the examination of acutely isolated SGCs, we also studied those that had been cultured for 3 days. Our results indicate that the gene expression profile of cultured peripheral glial cells changes significantly *in vitro*. We found that an entirely new population emerges upon culturing which we labelled “ *in vitro* glia”. It is characterized by expression of genes related to proliferation, expression of SGCs markers and a resemblance to Schwann cell precursors. In addition to these “ *in vitro* glia”, we also found that a proportion of cells within the “more physiological” SGC cluster in culture, change into a Schwann cell precursor-like state. This is in line with work from George *et al.*, who showed that long-term cultured SGCs have a similar transcriptional profile to that of long-term cultured Schwann cells ^26^. Our cultured cells were derived from two-week old mice, where the maturation of promyelinating Schwann cells to myelinating Schwann cells is still in process ^57^. We therefore cannot exclude that this developmental timeline for Schwann cells had an impact on our results.

The proliferation profile seen in the “ *in vitro* glia” is absent in acutely isolated SGCs. Specifically, at least transcriptionally, we did not find any evidence to suggest that adult SGCs cells are proliferating after nerve injury *in vivo*. Reports to the contrary ^9, 58– 60^ are confounded by the fact that they stained only for proliferation markers and attempted to identify SGCs by their position rather than by antibody staining. Especially after nerve injury, when macrophages closely approach SGC-neuron units, this intimate position of macrophages relative to the neuronal soma may easily be misinterpreted as SGCs when omitting detection of cellular markers ^13^. During development, SGCs and other cells do proliferate in the DRG, but this process has been shown to terminate around birth ^26, 61^. 

Like all single-cell studies, our analysis had limitations. Importantly, most current scRNA-seq experiments, including those presented here, rely on droplet-based technologies that are only able to detect a fraction of transcripts present in a given cell (~30%) ^62^. In the Cell_SNI dataset, we analysed 2565 SGCs, suggesting that across all SGCs, we are likely to have a good representation of the genes detectable in SGCs. Indeed, when we compiled all single cell transcripts to generate a pseudo-bulk profile, we found comparable expression to our own prior bulk sequencing results of sorted SGCs (see Extended Excel Sheet: SGC_gene_expression). Nevertheless, with either method, we may have missed very lowly expressed transcripts, like adhesion GPCRs (due to the number of cells analysed here, and the read depth used in 13).

Our differential expression analyses were generally rather variable – as indicated by the low number of commonly regulated genes identified across datasets. One possible explanation is the difference in time points and injury types. For instance, nerve crush is a regenerating model, while SNI is a chronic model causing persistent pain. Furthermore, the two datasets have been prepared with different dissociation strategies to obtain a single cell suspension prior to sequencing, which may also alter the expression of some genes ^63^. Another likely cause for the observed variability is that we were limited to performing the differential expression analyses on a cell-by-cell basis, an approach which lacks power and gives rise to a higher frequency of false positives. If we had had more biological replicates, we could have performed a pseudo-bulk analysis which might have shed further light on the common responses of SGCs to different nerve injuries ^64, 65^.

In conclusion, we found that SGCs share a common response following nerve crush and ligation, which includes regulation of genes involved in cholesterol biosynthesis. We also found that peripheral glial cells in culture change significantly, with many starting to resemble Schwann cell precursors. Our *in vitro* observations were in accordance with previous studies ^25, 26^ and emphasize how studies using SGC in a dish need to be approached and interpreted with caution.

## Data availability

### Underlying data

Web-based portal with user friendly interface:

Web-based portal at Broad Institute including the data from the used scRNAseq studies. https://singlecell.broadinstitute.org/single_cell/study/SCP1539/

The website includes processed data from Cell_SNI, Cell_Crush and Cell_culture datasets.

Data are available under the terms of the Creative Commons Zero “No rights reserved” data waiver (CC0 1.0 Public domain dedication.

Repository of raw data:

Gene Expression Omnibus: GSE139103 (Cell_Crush data), GSE174430 (Cell_SNI data) and GSE188971 (Cell_culture data). Data are available under the terms of the Creative Commons Zero “No rights reserved” data waiver (CC0 1.0 Public domain dedication).

### Extended data

Open Science Framework: Comparative transcriptional analysis of the satellite glial cell injury response. https://doi.org/10.17605/OSF.IO/J4DB2

This project contains the following extended data:

- Extended figures (PDF). Extended figure 1–5.
*Extended Figure 1:* Plot showing the cell types from the Cell_SNI dataset on the x-axis and the number of detected genes on the y-axis
*Extended Figure 2:* Heatmaps with the top 10 regulated neuronal genes plotted against the non-neuronal cell type clusters identified in each dataset. Black denotes that a gene is differentially regulated in the corresponding non-neuronal cluster and white that it is not regulated. SGC = satellite glial cells, VEC = vascular endothelial cells, VSMC = vascular smooth muscle cells and VECC = vascular endothelial capillary cells.
*Extended Figure 3:* SGCs clusters in Cell_SNI and Cell_crush datasets. A-B) UMAPS of the SGCs from the Cell_SNI dataset coloured based on clustering in A and injury condition in B. C-D) UMAPS of the SGCs from the Cell_crush dataset coloured based on clustering in C and injury condition in D.
*Extended Figure 4:* Overlap of the three different lists of 2000 variable genes determined from the gene expression of all SGCs. Disp = dispersion, Mvp = Mean.var.plot, Vst = Variance stabilizing transformation.
*Extended Figure 5:* Clustering of SGCs with the alternative methods. A-F) UMAPs of combined and integrated SGCs from the Cell_SNI (3153 cells) and Cell_crush (2147 cells) datasets. Analysis were performed with either dispersion method A-C or mean.var.plot D-F. A+D) UMAP coloured based on dataset. B+E) UMAP coloured based on clustering. Each number/colour denotes a cluster. C+F) UMAP coloured based on injury condition with 3321 SGCs from injured condition and 1979 SGCs from uninjured condition.
*Extended Figure 6:* STRING network of the regulated genes in the datasets. The Cell_SNI dataset is spilt based on timepoints. The genes are colour coded based on their annotation related to the 9 terms that both datasets had in common in Figure 2D. Gene names in bold indicate that the gene was upregulated after injury while italic gene names indicate that the gene was downregulated. The dashed line highlights the downregulated genes involved in the cholesterol biosynthesis pathway.
- Extended tables (PDF). Extended table 1–2
*Extended table 1:* Distribution of injured or naïve state of SGCs in the integrated clusters determined by the “vst” method (Figure 2A–C) or the alternative “Mean.var.plot” or “dispersion” methods (Extended Figure 5). Cell numbers are denoted in parenthesis.
*Extended table 2:* Distribution of the cell types in the joint analysis depending on the cells’ origin from either the Cell_SNI dataset (14 days, 7 days and naïve) or the Cell_culture dataset (Culture_A and Culture_B). The cluster analyses were performed with the “vst” method or the alternative “Mean.var.plot” or “dispersion” methods. Cell numbers are denoted in parenthesis.
- Extended Excel Sheet DE_analysis_metascape (XLSX): Differential expression of genes in SGCs after nerve injury in the Cell_SNI and Cell_crush datasets
- Extended Excel Sheet Renthal et al 7d (XLSX): Differential expression of genes in various cell types after nerve injury in the Renthal et al dataset at 7 days after injury.
- Extended Excel Sheet Wang et al 7d (XLSX): Differential expression of genes in various cell types after nerve injury in the Wang et al dataset at 7 days after injury
- Extended Excel Sheet SGC_gene_expression (XLSX): Comparison of number of expressed genes in SGCs in Cell_SNI dataset and Bulk RNAseq dataset.

Data are available under the terms of the Creative Commons Zero “No rights reserved” data waiver (CC0 1.0 Public domain dedication).
