# Supplementary material for: Comparative transcriptional analysis of satellite glial cell injury response
Source: Wellcome Open Res. 2022 May 19;7:156. [Version 1] doi: 10.12688/wellcomeopenres.17885.1 (PMC9329822; doi:10.12688/wellcomeopenres.17885.1)
Supplement: Peer review history

# Peer review: Reviewer response for version 1

**DOI:** 10.21956/wellcomeopenres.19811.r50916

**Published:** 2022-07-27

**Authors:** Kyle M. Baumbauer

**Affiliations:** 1 Department of Cell Biology and Physiology,, University of Kansas Medical Center, Kansas City, KS, USA

The ability of satellite glial cells (SGCs) to regulate primary afferent function under normal and injured conditions remains incompletely understood but is of utmost importance if we are to develop greater insight into the processes that regulate the emergence and (failure of) pain resolution. SGCs are notoriously difficult to study given their anatomic characteristics and lack of markers that uniquely identify them. Moreover, given the variety of methods used to study SGCs, and that all of the protocols used have the potential to differentially influence SGC profiles of gene and protein expression, it has been a challenge in the field to develop any consensus regarding SGC responses to injury or nociceptor activation. The current paper extends previous work by this group detailing the transcriptomic signature of SGCs in uninjured and nerve injured states. Here, the authors leveraged single cell RNA sequencing (scRNAseq) performed on SGCs from mice with sciatic nerve injury, as well as SGCs that have remained in culture for 3 days, and compared their results against publicly available sequencing datasets also containing SGC transcriptomic information.

 It isn’t clear why the Cell_culture experiment was performed with SWISS outbred mice when all other datasets being examined were obtained from C57Bl/6J inbred mice. The difference in the genetic background alone has a huge potential to influence transcriptional profile, and conclusions made regarding these data should be tempered.

 Is it possible that failure to detect an injury signature in SNI and nerve crush samples relative to naïve samples (using unsupervised clustering) is not just because transcriptional changes in the SGCs may be subtle, but that those subtle changes are being enriched against the background of dissection and cell dissociation which is a much more severe injury than SNI or nerve crush alone?

 There is a strong emphasis on *Gfap*, *Gja1*, and *Hmgcs1* not being differentially regulated above the preset detection thresholds. As the authors note, these targets are canonically used for identification of SGCs at the protein level, and given that the current data are from the transcriptional level with restricted timepoints, it is difficult to rule out that these genes are not being altered or that they are valid markers of SGCs without additional protein validation. Moreover, it is also possible that these genes/proteins are markers for individual populations of SGCs, and that the expression of these targets is dynamic over time and in response to injury. Furthermore, the authors describe results from previous reports demonstrating a loss of protein expression and reversion to a Schwann cell phenotype upon disconnection from their associated neuron. Is it possible that failure to detect the above genes represents the loss of proteins or reversion to a Schwann cell phenotype?

 Perhaps the most interesting finding is the differential regulation of genes involved in cholesterol biosynthesis ( *Idi1, Msmo1, Fdps, Fdft1, and Sqle*). The functional implication of these alterations has yet to be revealed, but it is implied by the authors that these genes are reliably expressed in SGCs. If this is true, does the clustering in Figures 3 and 4 change if cholesterol biosynthetic genes are used instead of more traditional SGC markers?

 The authors conclude that culturing SGCs leads to a reversion to a Schwann cell phenotype that is not observed in the Cell_SNI data. I think it is important to recognize that there are differences based on preparation/model methodology, as well as that dissociation of the DRG can be conceived as a severe injury that engages multiple repair programs that may confound analyses. This “reversion” may be part of this process following severe injury.

 Despite the noted issues, the authors provide additional data that make a significant contribution to our understanding of SGC biology.

Is the work clearly and accurately presented and does it cite the current literature?

Yes

If applicable, is the statistical analysis and its interpretation appropriate?

Yes

Are all the source data underlying the results available to ensure full reproducibility?

Yes

Is the study design appropriate and is the work technically sound?

Yes

Are the conclusions drawn adequately supported by the results?

Yes

Are sufficient details of methods and analysis provided to allow replication by others?

Yes

Reviewer Expertise:

Neuroscience, neurophysiology, nociceptor function, pain, inflammation, nerve injury

I confirm that I have read this submission and believe that I have an appropriate level of expertise to confirm that it is of an acceptable scientific standard.

# Peer review: Reviewer response for version 1

**DOI:** 10.21956/wellcomeopenres.19811.r50906

**Published:** 2022-06-24

**Authors:** Temugin Berta

**Affiliations:** 1 Pain Research Center, Department of Anesthesiology, University of Cincinnati Medical Center, Cincinnati, OH, USA

Pain is the primary reason why people seek medical care. In contrast to acute pain that is transient, chronic pain is a persistent, debilitating condition for which there are few treatment options. Currently, available pain drugs generally target neuronal pathways, but it ow clear that neurons are not the only players that drive the establishment and maintenance of chronic pain. Satellite glial cells (SGCs) tightly surround and support peripheral sensory neurons are emerging as novel therapeutic targets (Hanani and Spray, 2020 ^1^). And yet, SGC biology and functions are still incompletely known due to their morphology and a surprisingly lack in tools for their efficient investigation. In this manuscript, the authors leveraged advances in single-cell RNA sequencing (RNAseq) to investigate the transcriptome of SGCs and its alteration in neuropathic pain, a chronic pain condition caused by damage or injury of the nervous system. Using two animal models of neuropathic pain, namely the spared nerve injury and sciatic nerve crush models, the authors identified a common injury response of SGCs with an upregulation of several genes involved in cholesterol biosynthesis.

 This SGC response was also present in other single-cell and single-nucleus RNAseq datasets investigating neuropathic pain (Renthal et al., 2020 ^2^; Wang et al., 2021 ^3^), and regulation of genes involved in cholesterol biosynthesis have been recently associated with nerve regeneration (Avraham et al., 2020 ^4^). How and whether these genes are also associated with neuropathic pain is still unknown and the authors offer some speculations, but it is noteworthy to mention that nerve regeneration process has been linked to pain in various neuropathic pain models (Xie et al., 2020 ^5^). Interestingly, the authors also compared the transcriptomes of SGCs from their single-cell analysis to those from cell cultures, and observed substantial differences with cultured SGCs expressing genes associated with proliferation and indicating a regression to a Schwann cell precursor phenotype. This observation is important for future studies aiming to validate mechanisms and test drugs in cultured SGCs.

 Another interesting observation reported in the manuscript is the lack of transcriptional regulation of well-known SGC markers such as Connexin43 (Gja1) and GFAP (Gfap) in their single-cell analysis of neuropathic pain models. This leads us to some limitations of single-cell RNAseq analyses. First, RNAseq is limited to transcriptional analyses and generally does not consider potential post-transcriptional mechanisms and protein changes. As mentioned in the manuscript, increased expression of GFAP protein is often used as a marker for SGC reactivity by immunohistochemical analysis. Second, single-cell analyses require tissue dissociation that is prone to artifactual gene expression changes and can be optimized in future studies by the integration in the dissociation protocol of transcriptional inhibitors and lower temperatures (Marsh et al., 2022 ^6^). Third, single-cell RNAseq analyses suffer of shallow sequencing meaning that only a fraction of moderate to high-expressed genes are detected in a given cell, as recognized by the authors. Despite these limitations, the authors provide a very insightful overview of the injury response in SGCs, suggest caution in using cultured SGCs, and compiled the datasets they analyzed and made them easily accessible at the Broad Institute’s Single Cell Portal ( https://singlecell.broadinstitute.org/single_cell/study/SCP1539/).

Is the work clearly and accurately presented and does it cite the current literature?

Yes

If applicable, is the statistical analysis and its interpretation appropriate?

Yes

Are all the source data underlying the results available to ensure full reproducibility?

Yes

Is the study design appropriate and is the work technically sound?

Yes

Are the conclusions drawn adequately supported by the results?

Yes

Are sufficient details of methods and analysis provided to allow replication by others?

Yes

Reviewer Expertise:

Neuroscience

I confirm that I have read this submission and believe that I have an appropriate level of expertise to confirm that it is of an acceptable scientific standard.

## References

[1] : Emerging importance of satellite glia in nervous system function and dysfunction. *Nat Rev Neurosci* .21(9) : 10.1038/s41583-020-0333-z 485-498 10.1038/s41583-020-0333-z 32699292PMC7374656

[2] : Transcriptional Reprogramming of Distinct Peripheral Sensory Neuron Subtypes after Axonal Injury. *Neuron* .2020;108(1) : 10.1016/j.neuron.2020.07.026 128-144.e9 10.1016/j.neuron.2020.07.026 PMC759025032810432

[3] : Single-cell transcriptomic analysis of somatosensory neurons uncovers temporal development of neuropathic pain. *Cell Res* .31(8) : 10.1038/s41422-021-00479-9 904-918 10.1038/s41422-021-00479-9 33692491PMC8324866

[4] : Satellite glial cells promote regenerative growth in sensory neurons. *Nature Communications* .2020;11(1) : 10.1038/s41467-020-18642-y 10.1038/s41467-020-18642-y PMC752472632994417

[5] : Localized sympathectomy reduces peripheral nerve regeneration and pain behaviors in 2 rat neuropathic pain models. *Pain* .161(8) : 10.1097/j.pain.0000000000001887 1925-1936 10.1097/j.pain.0000000000001887 32701850PMC7572566

[6] : Dissection of artifactual and confounding glial signatures by single-cell sequencing of mouse and human brain. *Nat Neurosci* .25(3) : 10.1038/s41593-022-01022-8 306-316 10.1038/s41593-022-01022-8 35260865PMC11645269
